# Willingness to pay for short- and long-acting contraceptives among female adolescents and their parents in Brazil:a pilot study

**DOI:** 10.31744/einstein_journal/2021AO6376

**Published:** 2021-09-29

**Authors:** Daniela Farah, Manoel João Batista Castello Girão, Marcelo Cunio Machado Fonseca

**Affiliations:** 1 Universidade Federal de São Paulo São PauloSP Brazil Universidade Federal de São Paulo, São Paulo, SP, Brazil.

**Keywords:** Long-acting reversible contraception, Contraception, Adolescent, Brazil

## Abstract

**Objective:**

To evaluate willingness to pay for short- and long-acting reversible contraceptive methods among female Brazilian adolescents and their parents, as well as their perspective on using such methods.

**Methods:**

This is a cross-sectional study of female adolescents aged 13 to 19 years and their parents. We surveyed to estimate their willingness to pay for contraceptive methods. The values are expressed as mean±standard deviation in Brazilian reals (R$). Spearman correlation was employed for socioeconomic status of parents, age of adolescents and their standpoints. The methods types and adolescent and parent perspectives were analyzed by the test χ^2^. To determine an agreement between pairs and their willingness to pay, we used the Bland-Altman plot.

**Results:**

A total of 165 surveys were collected. Short-acting method was significantly more acceptable to pay than the long-action method, by both parents and their daughters. Parents and their daughters are willing to pay out-of-pocket R$ 52,25±22,48 and R$ 51,63±21,24 for short-acting reversible contraception method, and R$ 176,83±130,34 and R$ 174,83±143,64, for long-acting method, respectively. Bland-Altman analysis indicated an agreement on both perspectives and the price they are willing to pay for each contraceptive method.

**Conclusion:**

Parents and adolescent daughters are more willing to pay for short-acting methods. We showed an agreement between the parent and the daughter for the values paid for each method.

## INTRODUCTION

Long-acting reversible contraception (LARC) methods do not rely on daily adherence, showing better effectiveness than short-acting reversible contraception (SARC) methods.^(1)^ However, there is relatively low uptake of these methods, partly due to their quite high initial cost.^(2)^ Another obstacle is that LARC methods require procedures performed by healthcare professionals, and their provision may take longer. Also, there is a lack of information regarding these methods.^(2)^

In the United States, approximately 4.3% of adolescents using a contraceptive method choose LARC.^(3)^ A cross-sectional study performed in Brazil showed only 2.4% of entire cohort were using LARC and, interestingly, 58.7% of women who had never used the intrauterine device (IUD) would still not use it.^(4)^

A nationwide survey taken in Sweden showed 11% of all contraception users reported they would use another method if they had the same price. An implant was the most desired contraceptive method among these women.^(5)^ The CHOICE project showed the preferred choice of adolescents would be LARC methods over SARC, when some barriers were removed, such as lack of information, cost, and difficulty getting access.^(6)^ One study explored reducing cost barriers of LARC methods to increase the access and demonstrated increased LARC uptake.^(7)^

The use of LARC may have a potential role in reducing unintended pregnancies, because they are safe, effective, user-independent, and associated with high levels of user satisfaction.^(6)^

We hypothesized the main problem of underuse of LARC is related to high up-front cost and lack of information. We also speculated parents would be more willing for their daughters to use LARC than the adolescents.

## OBJECTIVE

To evaluate willingness of Brazilian female adolescent and their parents to pay for short- and long-acting reversible contraceptive methods, and their perspective on use of the methods.

## METHODS

This is a cross-sectional survey with female adolescents aged 13 to 19 years, and one of their parents were required to answer a questionnaire built in platform Survey Monkey Inc^®^ (San Mateo, California, USA) to be eligible for the study. Initially, the parents had to approve the daughter’s participation in the research. The data were analyzed in pairs (parent/daughter). All participants gave their written informed consent from before application of the questionnaire.

We presented an introductory movie to all participants before application of the questionnaire. The purpose of the movie was to present all contraceptive choices available in Brazil, and their pros and cons.

We performed data collection during April and June 2018, in São Paulo (SP), Brazil. The questionnaire collected data on the adolescent and their parent standpoints, separately. For each participant, we asked about acceptance of LARC and SARC, and their willingness to pay for both classes of contraceptive methods. The payment could be out-of-the-pocket or subsidized for both classes of contraception.

**Figure 1:**
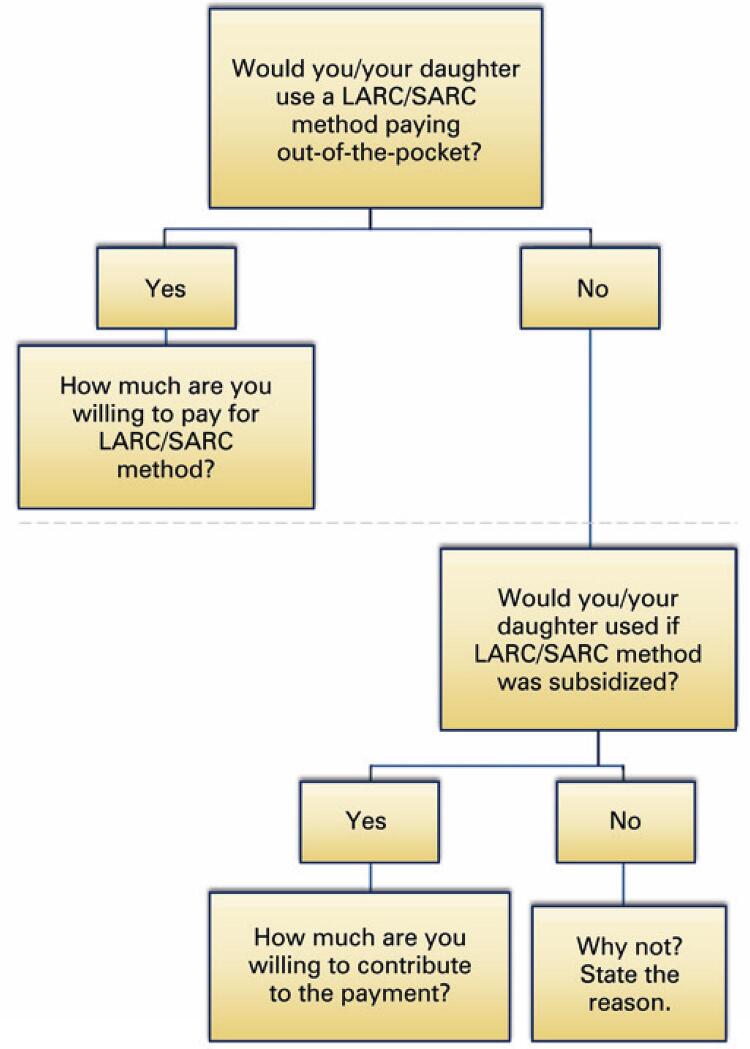
Survey flowchart

LARC: long-acting reversible contraception; SARC: short-acting reversible contraception.

We included the following variables in the survey: age of the female adolescent, socioeconomic status of the family, willingness-to-pay for LARC and SARC methods. To assess willingness to pay, we first asked if they agreed to use one of the classes of contraception (LARC or SARC), by paying out-of-the-pocket; if affirmative, how much were they willing to pay for the method (Figure 1). If negative, we secondly asked about acceptance of the method, if it was subsidized. If affirmative, we asked how much they were willing to contribute to the method. If negative, we asked to state the reason for not accepting (Figure 1). These steps were performed for both classes of contraception, individually.

The currency to assess willingness to pay was Brazilian reals (R$). The conversion rate for US dollars (US$) for the year 2018 is US$ 1.00=R$ 3.6552.

To analyze the use of LARC and SARC between parent and daughter, we used the Fisher exact test. We employed the Spearman correlation for socioeconomic status and the parent’s willingness to pay, and the adolescent willingness to pay and their age using PAST version 3.25 software. We prepared the Bland-Altman graph to identify the agreement between the values of willingness to pay for both classes of contraceptive methods among parents and daughters. We conducted this analysis in pairs of parents and daughters, who had decided to pay any value for the contraceptive methods. We calculated descriptive statistics using mean±standard deviation.

We obtained ethical approval from the institutional review board of the *Universidade Federal de São Paulo* (UNIFESP), CAAE: 58281316.4.0000.5505, protocol 2.032.537.

## RESULTS

A total of 174 questionnaires were answered, but nine parents did not authorize their daughters to answer. Therefore, we included a total of 165 questionnaires in this analysis. Most adolescent (67%) were aged 18 and 19 years, mean age of 17.6±1.4 years. During the survey, 55% of adolescents reported not using any contraceptive method. Among the adolescents that were using a contraceptive method, 81% stated that they were taking a birth control pill. Most participants (70%) had a monthly income of two to four minimum-wages.

More than 70% of both parents and daughters said they were aware of contraceptive methods (73% and 71%, respectively). Approximately 80% of survey participants responded adequately to the video questions, demonstrating a good understanding of the subject (79% for parents and 82% for adolescents).

**Figure 2:**
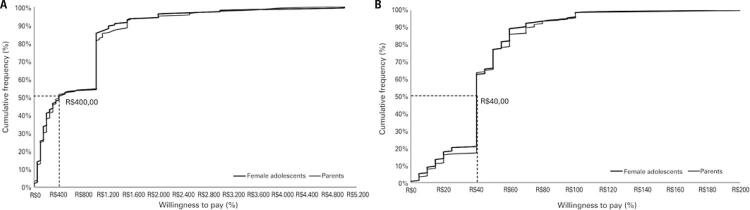
Cumulative frequency of parents and adolescent willing to pay. A) Long-acting reversible contraception method; B) Short-acting reversible contraception method

LARC: long-acting reversible contraception; SARC: short-acting reversible contraception.

The acceptance rate of LARC methods was 44% among parents (72 out of 165 parents) and 42% among adolescents (69 out of 165 adolescents) who were willing to pay out-of-pocket. Out of the remaining parents (56%) and adolescents (58%), 88% of parents would let their daughter use a LARC method, if it were subsidized, and 83% of adolescents would use if it were subsidized. The values expressed for willingness to pay are in table 1. The median willingness to pay for a LARC method was R$ 400,00, for both parents and daughters (Figure 2A).

**Table 1:** Willingness-to-pay values for parents and adolescents

	Parents		Adolescent girls	
	
	Out-of-pocket (R$)	Subsidized (R$)	Out-of-pocket (R$)	Subsidized (R$)
LARC, (%)	1.377,78±792,30 (44)	176,83 ±130,34 (49)	1.348,55±842,50 (42)	174,83±143,64 (48)
SARC, (%)	52,25±22,48 (79)	13,46±6,45 (16)	51,63±21,24 (76)	13,75±7,18 (20)

Results expressed as mean±standard deviation.

LARC: long-acting reversible contraception; SARC: short-acting reversible contraception.

Those parents who did not accept a LARC method, even if it were available for free, 12% indicated lack of information and uncertainties about safety of LARC methods as the reasons for their denial. The adolescents who alleged they would not use a LARC method, even if it were for free (17%), stated the main reasons would be lack of information, fear about the safety, and one adolescent declared she would not use due to the long time the device would be in place.

The acceptance rate of SARC methods was 79% among parents (131 out of 165 parents) and 76% among adolescents (126 out of 165 adolescents) that were willing to pay out-of-pocket. For the remaining parents (21%) and adolescents (24%), 76% of parents would let their daughter use a SARC method, if it were subsidized, and 81% of adolescents would use, if it were subsidized. The values expressed for willingness to pay are in table 1. The median willingness to pay for a SARC method was R$ 40,00 for both parents and daughters (Figure 2B).

Only eight parents (24%) said they would not allow their daughters to use a SARC method even for free, and the main reason was the daily use of hormonal contraceptives. Moreover, only seven girls (18%) said that they would not use a SARC method even if it were free. The main reasons were their dislike of hormonal contraception, the need for daily adherence, or simply not desiring to use a contraceptive method.

**Figure 3:**
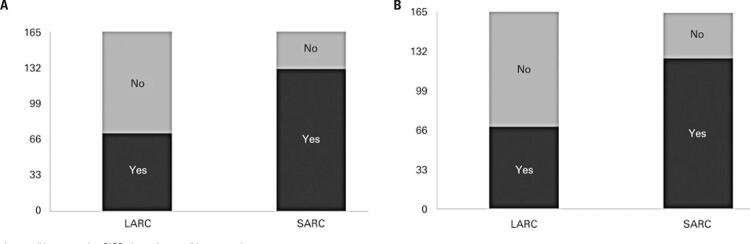
First acceptance of each class of contraceptive method (out-of-pocket). A) Parent standpoint; B) Daughter standpoint

LARC: long-acting reversible contraception; SARC: short-acting reversible contraception.

The acceptance of SARC, when compared to LARC methods, was preferred by parents and adolescents when paid out-of-pocket (p_parents_<0.0001 and p_adolescents_<0.0001) (Figures 3A and 3B).

However, the proportion of parents who did not allow contraception, under any circumstance, was not different between LARC and SARC methods (12% *versus* 24%, respectively; p=0.157). There was a weak correlation regarding the parents’ socioeconomic level and the value they would be willing to pay for both LARC and SARC methods (r_s_=0.382 and r_s_=0.326, respectively).

Likewise, the proportion of adolescents that stated would not use a contraception method under any circumstances, was not different between the LARC and SARC methods (17% *versus* 18%, respectively; p=0.857). There was no correlation regarding the adolescent age and the value expressed, both in LARC and SARC methods (r_s_=-0.128 and r_s_=-0.053, respectively).

We did not observe any difference in the uptake of LARC and SARC, between parents and the daughters, neither for out-of-pocket (p_LARC_=0.824 and p_SARC_=0.595) nor for subsidized (p_LARC_=0.410 and p_SARC_=0.576).

**Figure 4:**
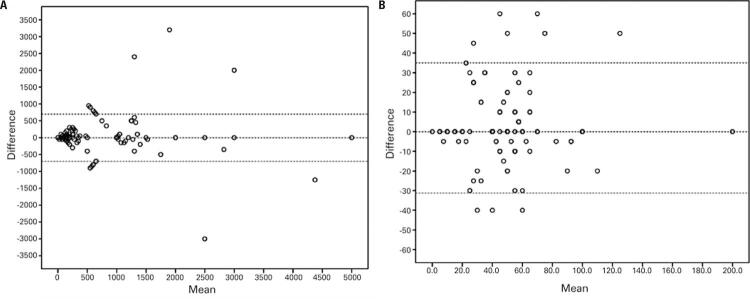
. Bland-Altman plot for agreement between parents and daughters. A) Long-acting reversible contraception method; B) Short-acting reversible contraception method

The Bland-Altman analysis comprised 147 pairs and 156 pairs for LARC and SARC, respectively. This analysis showed an agreement between parents and daughters for the values they were willing to pay (Figures 4A and 4B).

## DISCUSSION

This study is the first to assess the willingness of adolescents and their parents to pay for contraception methods. We raised the hypothesis that, in Brazil, parents would be willing to pay more than their daughters for the contraceptive methods. We could not prove it, because counteracting our hypothesis, both adolescents and parents provided similar values of how much they were willing to pay. Moreover, parents and adolescents were more comfortable to accept out-of-pocket SARC rather than LARC methods. We were also able to show that a low percentage of both parents and adolescents would not use any form of contraception, even if it were available for free.

The use of contraception by young women is the best strategy to prevent unintended pregnancy and should be encouraged by governments as a public health policy. Several entities recommend using LARC as the best contraceptive choice for adolescents and young women, since they are safe and more efficient methods.^(8-11)^ However, there are still some issues and myths majorly regarding safety of these methods, creating barriers to disseminating them among adolescents. Our study showed the most cited reason for not using a LARC method was safety, and both parents and daughters mentioned it.

A survey conducted in the United States identified the patients’ most cited myths and misconceptions about LARC methods, including they caused menstrual irregularities, weight gain, increased risk of cancers, and ectopic pregnancy.^(12,13)^ Regarding IUD only, the list is long, including abortions, pelvic inflammatory disease, infertility, and too big to be placed in nulliparous women.^(12)^ We speculate these misconceptions and myths are raised by their next of kin, and this hypothesis may be sustained by the agreement between parents and their daughters regarding the value they would pay for both methods. Hence, we agree with numerous specialists that education is a key factor to be extensively approached by health care professionals, to promote a more valuable and better choice for adolescents.^(2,14)^

Our study showed the acceptance of SARC methods by both parents and daughters is higher than LARC methods, when the decision is to pay out-of-pocket. Indeed, a study conducted in Spain also showed that young women, aged up to 29 years, preferred to use SARC methods, such as combined oral contraceptive, vaginal ring, and male condom, for contraception.^(15)^ This result highlights the importance of broader education and easier access to LARC methods to the population, especially young women, to prevent unintended pregnancies.

The main reason adolescents did not desire the SARC method was the daily hormonal dose and the consequent safety issue of increasing the risk of thrombosis. Indeed, combined oral contraceptives, popularly known as birth control pills, may cause thrombosis. However, some studies alert some other risk factors that further increase this risk, such as obesity, smoking habit, and family history.^(16)^ One study conducted in Japan identified that this risk is more likely to occur in women aged over 40 years.^(17)^

Our study has some limitations. First, this is a pilot study focusing on the Brazilian population’s willingness to pay for contraceptive methods used by adolescents; therefore, it has no statistical power. Second, since the Brazilian Public Health System (SUS - *Sistema Único de Saúde*) only supplies the copper IUD as LARC, there is scarce knowledge about such method. Nonetheless, we have presented an introductory movie about all contraceptive methods. Third, we did not obtain a correlation between the socioeconomic status and the value the participants were willing to pay for the methods, due to the lack of representativeness of the different socioeconomic brackets. Most participants were from the lower socioeconomic brackets. Fourth, we employed the questionnaire simultaneously for parents and daughters; this approach may have limited the number of adolescent respondents and influenced their decision.

An educational approach for both adolescents and parents about the contraceptive options is of utmost importance for the former, especially at the beginning of their sexual life. Implementing programs and campaigns to raise awareness on sexual and family planning to these populations are primordial, along with a more comprehensive availability of LARC methods. These actions can decrease unintended pregnancy in adolescents, and reduce health and social consequences, such as maternal death, prematurity, childhood morbidity, and school dropouts.

## CONCLUSION

A higher percentage of parents and their daughters showed a greater acceptance of short-acting reversible contraception methods, when asked to pay out-of-pocket, as compared to long-acting reversible contraceptive methods. A low percentage of participants would not use at all any form of contraception. The values they were willing to pay for long-acting or short-acting reversible contraception methods did not correlate with socioeconomic status or adolescent age. We found an agreement for the value parents and daughters were willing to pay for long- and short-acting reversible contraceptive methods.
