# Supplementary material for: Willingness to pay for short- and long-acting contraceptives among female adolescents and their parents in Brazil:a pilot study
Source: Einstein (Sao Paulo). 2021 Sep 29;19:eAO6376. doi: 10.31744/einstein_journal/2021AO6376 (PMC8483636; doi:10.31744/einstein_journal/2021AO6376)
Supplement: Translation

# Translation: Disposição de mulheres adolescentes e seus pais a pagar por contraceptivos de curta e longa duração no Brasil: estudo-piloto

**Authors:** Daniela Farah, Manoel João Batista Castello Girão, Marcelo Cunio Machado Fonseca

**Affiliations:** 1 Brasil Universidade Federal de São Paulo, São Paulo, SP, Brasil.

**Keywords:** Contracepção reversível de longo prazo, Anticoncepção, Adolescente, Brasil

## Abstract

**Objetivo:**

Avaliar a disposição de mulheres adolescentes e seus pais a pagar por métodos contraceptivos reversíveis de longa e curta duração, além de suas perspectivas ao usá-los.

**Métodos:**

Este é um estudo transversal com adolescentes de 13 a 19 anos e seus pais. Foi feita uma pesquisa para estimar a disposição a pagar por métodos anticoncepcionais. Os valores estão expressos em média±desvio-padrão em Reais (R$). Foi feita a correlação de Spearman para nível socioeconômico dos pais, idade das adolescentes e seus pontos de vista. Os tipos de métodos e as perspectivas de adolescentes e pais foram analisados pelo teste χ^2^. Para determinar a concordância entre os pares e sua disposição a pagar, foi usado o gráfico de Bland-Altman.

**Resultados:**

Foram coletadas 165 pesquisas. O método de curta duração foi significativamente mais aceitável para pagar que o de longa duração, por ambos pais e filhas. Os pais e suas filhas estavam dispostos a pagar, do próprio bolso, R$ 52,25±22,48 e R$ 51,63±21,24, pelo método contraceptivo reversível de curta duração, e R$ 176,83±130,34 e R$ 174,83±143,64, pelo de longa duração, respectivamente. A análise de Bland-Altman indicou concordância sobre ambas as perspectivas e o preço a que estavam dispostos a pagar por cada método anticoncepcional.

**Conclusão:**

Pais e adolescentes estão mais dispostos a pagar pelos métodos reversíveis de curta duração. Houve concordância entre o pai e a filha para os valores pagos por cada método.

## INTRODUÇÃO

Os métodos contraceptivos reversíveis de longa duração (LARCs - *long-acting reversible contraceptive*) não dependem da adesão diária e mostram melhor efetividade que os métodos contraceptivos reversíveis de curta duração (SARC - *short-acting reversible contraception*).^(1)^Entretanto, há uma utilização relativamente baixa desses métodos, em parte por conta do custo inicial alto.^(2)^ Outro obstáculo é que os métodos LARCs requerem procedimentos realizados por profissionais de saúde e sua provisão pode levar mais tempo. Além disso, há uma falta de informação a respeito de tais métodos.^(2)^

Nos Estados Unidos, aproximadamente 4,3% das adolescentes que utilizam um método contraceptivo escolhem o LARC.^(3)^ Estudo transversal realizado no Brasil mostrou que apenas 2,4% de toda a coorte utilizava o LARC, e, curiosamente, 58,7% das mulheres que nunca tinham utilizado o dispositivo intrauterino (DIU) continuariam a não usar.^(4)^

Pesquisa nacional realizada na Suécia mostrou que 11% de todos os usuários de contracepção relataram que utilizariam outro método se todos tivessem o mesmo preço. Um implante era o método contraceptivo mais desejado entre essas mulheres.^(5)^ O projeto CHOICE mostrou que a escolha preferida das adolescentes seria por métodos LARCs em relação ao SARC, quando fossem removidas algumas barreiras, como falta de informações, custo e dificuldade de acesso.^(6)^ Um estudo explorou diminuir as barreiras de custo dos métodos LARCs para aumentar o acesso e demonstrou maior utilização deles.^(7)^

O uso de LARCs pode ter papel potencial na redução de gravidez indesejada, pois são seguros, eficazes, independentes do usuário e associados a altos níveis de satisfação das usuárias.^(6)^

Levantou-se a hipótese de que o principal problema da subutilização do LARC está relacionado com o alto custo inicial e a falta de informações. Também especulou-se que os pais estariam mais dispostos a ter as filhas usando LARC do que as próprias adolescentes.

## OBJETIVO

Avaliar a disposição de adolescentes brasileiras e seus pais a pagar por métodos contraceptivos reversíveis de longa duração e de curta duração e suas perspectivas sobre o uso deles.

## MÉTODOS

Esta é uma pesquisa transversal com adolescentes de 13 a 19 anos, e um de seus pais foi solicitado a responder um questionário integrado à plataforma SurveyMonkey Inc.^®^ (San Mateo, Califórnia, Estados Unidos) para ser elegível para o estudo. Inicialmente, os pais tiveram que aprovar a participação da filha na pesquisa. Os dados foram analisados em pares (pai/mãe/filha). Todos os participantes deram consentimento por escrito, antes da aplicação do questionário.

Apresentou-se um filme introdutório a todos os participantes antes da aplicação do questionário. O propósito do filme era expor todas as escolhas contraceptivas disponíveis no Brasil, bem como seus prós e contras.

A coleta de dados foi realizada entre abril e junho de 2018, em São Paulo (SP). O questionário coletou dados sobre os pontos de vista da adolescente e de seus pais, separadamente. Para cada participante, foi perguntado sobre a aceitação de LARC e SARC e sobre sua disposição a pagar pelas duas classes de métodos contraceptivos. O pagamento poderia ser pago de próprio bolso ou subsidiado para ambas as classes de contracepção.

Foram incluídas as seguintes variáveis na pesquisa: idade da adolescente, condição socioeconômica da família e disposição a pagar pelos métodos LARC e SARC. Para avaliar a disposição a pagar, primeiramente, foi perguntado se concordariam em usar uma das classes de contracepção (LARC ou SARC) pagando com recursos financeiros próprios; se afirmativo, quanto estariam dispostos a pagar pelo método (Figura 1). Se negativo, perguntou-se, em segundo lugar, se o método fosse subsidiado; se afirmativo, perguntou-se quanto eles estariam dispostos a contribuir para o método. Se negativo, foi questionado o motivo da não aceitação (Figura 1). Essas etapas foram realizadas para ambas as classes de contracepção, individualmente.

**Figura 1:** Fluxograma da pesquisa

LARC: contraceptivo reversível de longa duração; SARC: contraceptivo reversível de curta duração.

A moeda para avaliar disposição a pagar foi o Real (R$). A taxa de conversão para dólares americanos (US$) para o ano de 2018 foi US$ 1,00=R$ 3,6552.

Para analisar o uso de LARC e SARC entre pai e filha, foi usado o teste exato de Fisher. Empregou-se a correlação Spearman para analisar a condição socioeconômica e a disposição dos pais a pagar; a disposição da adolescente a pagar e sua idade foram verificadas pelo software PAST, versão 3.25. Foi feito o gráfico Bland-Altman para identificar a concordância entre os valores da disposição a pagar por ambas as classes de métodos contraceptivos entre pais e filhas. Foi conduzida esta análise em pares de pais e filhas, que decidiram pagar qualquer valor pelos métodos anticoncepcionais. Calculou-se a estatística descritiva usando média±desvio padrão.

Foi obtida aprovação ética do conselho de revisão institucional da Universidade Federal de São Paulo (UNIFESP), CAAE: 58281316.4.0000.5505, parecer 2.032.537.

## RESULTADOS

No total, 174 questionários foram respondidos, mas nove pais não autorizaram as filhas a responder. Dessa forma, 165 questionários foram incluídos neste estudo. A maioria das adolescentes (67%) tinha 18 e 19 anos, e a média de idade foi de 17,6±1,4 anos. No momento da pesquisa, 55% das meninas adolescentes relataram não utilizar nenhum método contraceptivo. Entre as adolescentes que usavam um método contraceptivo, 81% afirmaram que tomavam pílula anticoncepcional. A maioria das participantes (70%) tinha nível de renda mensal entre dois e quatro salários mínimos.

Mais de 70% dos pais e filhas disseram conhecer os métodos contraceptivos (73% e 71%, respectivamente). Aproximadamente 80% dos participantes da pesquisa responderam adequadamente às perguntas em vídeo, demonstrando boa compreensão do assunto (79% para pais e 82% para adolescentes).

A taxa de aceitação dos métodos LARCs foi de 44% entre os pais (72 de 165 pais) e 42% entre as adolescentes (69 de 165 adolescentes) dispostos a pagar com recursos próprios. Dentre os outros pais (56%) e adolescentes (58%), 88% dos pais deixariam sua filha usar um método LARC, se fosse subsidiado, e 83% das adolescentes usariam, também caso fosse subsidiado. Os valores expressos para a disposição a pagar estão na tabela 1. A disposição mediana a pagar por um método LARC foi de R$ 400,00, tanto para pais quanto para filhas (Figura 2A).

**Tabela 1:** Valores de disposição a pagar para pais e adolescentes

	Pais		Adolescentes	
	
	Pagamento do próprio bolso (R$)	Subsidiado (R$)	Pagamento do próprio bolso (R$)	Subsidiado (R$)
LARC, (%)	1.377,78±792,30 (44)	176,83 ±130,34 (49)	1.348,55±842,50 (42)	174,83±143,64 (48)
SARC, (%)	52,25±22,48 (79)	13,46±6,45 (16)	51,63±21,24 (76)	13,75±7,18 (20)

Resultados expressos com média±desvio padrão.

LARC: contraceptivo reversível de longa duração; SARC: contraceptivo reversível de curta duração.

**Figura 2:** Frequência acumulada de pais e adolescentes dispostos a pagar. A) Contraceptivo reversível de longa duração; B) Contraceptivo reversível de curta duração

LARC: contraceptivo reversível de longa duração; SARC: contraceptivo reversível de curta duração.

Dos pais que não aceitaram um método LARC, mesmo que disponível gratuitamente, 12% indicaram a falta de informações e as incertezas sobre a segurança dos métodos LARCs como as razões para sua negação. Aquelas adolescentes que alegaram que não usariam um método LARC, mesmo que fosse gratuito (17%), afirmaram que as principais razões seriam falta de informação e temor sobre a segurança; uma adolescente declarou que não usaria devido ao longo tempo durante o qual o dispositivo ficaria instalado.

A taxa de aceitação dos métodos SARCs foi de 79% entre os pais (131 de 165 pais) e 76% entre as adolescentes (126 de 165 adolescentes), que estavam dispostos a pagar do próprio bolso. Para os demais pais (21%) e adolescentes (24%), 76% dos pais deixariam sua filha usar um método SARC se fosse subsidiado, e 81% das adolescentes usariam, caso fosse subsidiado. Os valores expressos para disposição a pagar estão na tabela 1. A disposição mediana a pagar por um método SARC foi de R$ 40,00 para pais e filhas (Figura 2B).

Apenas oito pais (24%) disseram que não permitiriam que suas filhas usassem um método SARC, mesmo se fosse gratuito, e a principal razão era o uso diário de contraceptivos hormonais. Além disso, apenas sete meninas (18%) disseram que não utilizariam um método SARC, mesmo que fosse gratuito. As principais razões foram sua aversão à contracepção hormonal, a necessidade de adesão diária ou simplesmente o desejo de não usar um método contraceptivo.

A aceitação do SARC, quando comparado aos métodos LARCs, foi preferida pelos pais e pelas adolescentes quando pagos dos próprios bolsos (p_pais_<0,0001 e p_adolescentes_<0,0001) (Figuras 3A e 3B).

**Figura 3:** Primeira aceitação de cada classe de método contraceptivo (pagamento do próprio bolso). A) Ponto de vista do pai; B) Ponto de vista da filha

LARC: contraceptivo reversível de longa duração; SARC: contraceptivo reversível de curta duração.

Entretanto, a proporção de pais que não permitiriam a contracepção em nenhuma circunstância não foi diferente entre os métodos LARC e SARC (12% *versus* 24%, respectivamente; p=0,157). Houve fraca correlação com a condição socioeconômica dos pais e o valor a que estariam dispostos a pagar por ambos os métodos LARC e SARC (r_s_=0,382 e r_s_=0,326, respectivamente).

Da mesma forma, a proporção de adolescentes que afirmaram que não usariam um método contraceptivo em nenhuma circunstância não foi diferente entre os métodos LARC e SARC (17% *versus* 18%, respectivamente; p=0,857). Não houve correlação em relação à idade das meninas adolescentes e o valor expresso, tanto nos métodos LARC e SARC (r_s_=-0,128 e r_s_=-0,053, respectivamente).

Não foi observada diferença na utilização de LARC e SARC, entre pais e filhas, nem para pagar do próprio bolso (p_LARC_=0,824 e p_SARC_=0,595), nem para ser subsidiado (p_LARC_=0,410 e p_SARC_=0,576).

A análise Bland-Altman compreendeu 147 pares e 156 pares para LARC e SARC, respectivamente. Esta análise mostrou concordância entre pais e filhas para os valores que estavam dispostos a pagar (Figuras 4A e 4B).

**Figura 4:** Gráfico de Bland-Altman para concordância entre pais e filhas. A) Contraceptivo reversível de longa duração; B) Contraceptivo reversível de curta duração

## DISCUSSÃO

Este estudo é o primeiro a avaliar a disposição de adolescentes e de seus pais a pagar por métodos contraceptivos. Foi levantada a hipótese de que, no Brasil, os pais estariam dispostos a pagar mais pelos métodos anticoncepcionais do que suas filhas. Não foi possível prová-la, pois, contrariando esta hipótese, tanto as adolescentes quanto os pais informaram valores semelhantes para quanto estavam dispostos a pagar. Além disso, ambos, pais e filhas, sentiam-se mais à vontade para aceitar os métodos SARCs, pagando do próprio bolso, do que os LARCs. Também foi possível mostrar que uma baixa percentagem de pais e adolescentes não usaria nenhuma forma de contracepção, mesmo que esta estivesse disponível gratuitamente.

O uso de contracepção por mulheres jovens é a melhor estratégia para prevenir gravidez indesejada e deve ser encorajado pelos governos como uma política de saúde pública. Várias entidades recomendam o uso de LARC como a melhor opção contraceptiva para adolescentes e mulheres jovens, já que são métodos seguros e mais eficientes.^(8-11)^ Entretanto, ainda existem algumas questões e alguns mitos, principalmente em relação à segurança desses métodos, os quais criam barreiras para disseminá-los entre as adolescentes. Este estudo mostrou que a razão mais citada para não utilizar um método LARC foi segurança, mencionada tanto por pais e quanto por filhas.

Pesquisa realizada nos Estados Unidos identificou os mitos e conceitos errôneos mais citados pelos pacientes sobre os métodos LARCs, incluindo que causavam irregularidades menstruais, ganho de peso, maior risco de câncer e gravidez ectópica.^(12,13)^ Em relação apenas ao DIU, a lista é longa, incluindo abortos, doença inflamatória pélvica, infertilidade e ser grande demais para ser colocado em nulíparas.^(12)^Especulou-se que tais conceitos errôneos e mitos sejam levantados por seus parentes mais próximos, e essa hipótese pode ser sustentada pela concordância entre os pais e as filhas quanto ao valor que pagariam por ambos os métodos. Portanto, concorda-se com diversos especialistas de que a educação é um fator essencial a ser amplamente abordado pelos profissionais de saúde, de modo a promover uma escolha mais valiosa e melhor para as adolescentes.^(2,14)^

Este estudo mostrou que a aceitação dos métodos SARCs tanto pelo pai como por suas filhas é maior do que dos métodos LARCs, quando a decisão é paga do próprio bolso. De fato, estudo realizado na Espanha também mostrou que mulheres jovens, de até 29 anos, preferiram usar métodos SARCs, como contraceptivos orais combinados, anel vaginal e preservativo masculino, para contracepção.^(15)^ Esse resultado destaca a importância de uma educação mais ampla e de um acesso mais fácil aos métodos LARCs para a população, especialmente mulheres jovens, para evitar a gravidez não planejada.

As principais razões de as adolescentes não desejarem o método SARC foram a dose hormonal diária e a consequente questão de segurança, por aumentar o risco de trombose. De fato, os contraceptivos orais combinados, popularmente conhecidos como pílulas anticoncepcionais, podem causar trombose. Entretanto, alguns estudos alertam que alguns outros fatores de risco aumentam ainda mais esse risco, como obesidade, tabagismo e histórico familiar.^(16)^ Um estudo realizado no Japão identificou que tal risco é mais provável de ocorrer em mulheres com mais de 40 anos.^(17)^

Este estudo tem algumas limitações. Primeiramente, é um estudo-piloto focado na disposição da população brasileira a pagar por métodos contraceptivos usados por adolescentes, ou seja, não tem poder estatístico. Segundo, como o Sistema Único de Saúde (SUS) só fornece o DIU de cobre como LARC, o conhecimento sobre tais métodos é escasso. Porém, foi apresentado um filme introdutório sobre todos os métodos contraceptivos. Em terceiro lugar, não foi obtida correlação entre a condição socioeconômica e o valor que os participantes estavam dispostos a pagar pelos métodos, devido à falta de representatividade das diferentes classes socioeconômicas. A maioria dos participantes era de classes socioeconômicas mais baixas. Em quarto lugar, o questionário foi aplicado simultaneamente para pais e filhas; esta abordagem pode ter limitado o número de adolescentes que responderam e influenciado em sua decisão.

Uma abordagem educacional, tanto para as adolescentes quanto para os pais, sobre as opções contraceptivas é de suma importância para as primeiras, especialmente no início de sua vida sexual. A implementação de programas e campanhas de conscientização sobre planejamento sexual e familiar para essas populações é primordial, juntamente de uma disponibilidade mais abrangente dos métodos LARCs. Essas ações reduzem a gravidez não planejada na adolescência e as consequências de saúde e sociais, como morte materna, prematuridade, morbidade infantil e abandono escolar.

## CONCLUSÃO

Uma percentagem mais elevada de pais e suas filhas mostrou maior aceitação de métodos contraceptivos reversíveis de curta duração, quando solicitada a pagar do próprio bolso, em comparação aos de longa duração. Uma baixa percentagem de participantes não usaria nenhuma forma de contracepção. Os valores que estariam dispostos a pagar por métodos contraceptivos reversíveis de longa ou curta duração não estavam correlacionados com a condição socioeconômica ou idade das adolescentes. Houve concordância quanto ao valor que pais e filhas estavam dispostos a pagar por métodos anticoncepcionais reversíveis de longa e curta duração.
